# Supplementary material for: Zearalenone-14-Glucoside Is Hydrolyzed to Zearalenone by β-Glucosidase in Extracellular Matrix to Exert Intracellular Toxicity in KGN Cells
Source: Toxins (Basel). 2022 Jul 4;14(7):458. doi: 10.3390/toxins14070458 (PMC9316695; doi:10.3390/toxins14070458)
Supplement: Supplementary file 1 [file toxins-14-00458-s001.zip › toxins-1768971-supplementary.pdf]

toxins

MDPI

Article

Zearalenone-14-Glucoside Is Hydrolyzed to Zearalenone by β-Glucosidase in Extracellular Matrix to Exert Intracellular Toxicity in KGN cells

Haonan Ruan, Yunyun Wang, Yong Hou, Jing Zhang, Jiashuo Wu, Fangqing Zhang, Ming Sui, Jiaoyang Luo * and Meihua Yang *

1. Materials and Methods

1.1. Accuracy and precision

Both accuracy and precision were assessed using spiked samples. The accuracy of the proposed method was evaluated by the recoveries (n = 6), which were calculated using the following equation: % Recovery= (Area spiked sample – Area unspiked sample)/ Area standard×100%. Intra-day (n = 6) and inter day precision (n = 6) for each mycotoxins were assessed on one day and on three continuous days and were evaluated based on relative standard deviation (RSD) values.

2. Results

Table S1. Optimized MS/MS Parameter of ZEN, Z14G, α-ZEL and β-ZEL.

Mycotoxin	Ionization Mode	Q1	Species	Q2	Rt	DP	CE	CXP
ZEN	ESI^-	317.0	$[M-H]^-$	175.1*	5.1	−100	−42	−7
				131.1			−40	−25
Z14G	ESI^+	479.2	$[M-H]^+$	283.4*	2.7	110	20	15
				319.2			20	18
α-ZEL	ESI^-	319.3	$[M-H]^-$	159.9*	4.4	−140	−40	−13
				174.3			−34	−8
β-ZEL	ESI^-	319.2	$[M-H]^-$	275.3*	3.9	−95	−34	−9
				301.3			−32	−10

Q1:Precursor ion; Q2:Product ions; Rt: Retention time; DP: Declustering potential; CE: Collision energy; CXP: cell exit potential; *, quantitative ion.

Table S2. Accuracy and Precision of ZEN,Z14G, α-ZEL and β-ZEL.

Mycotoxins	Intra-day Precision (RSD%, n = 6)	Inter-day Precision (RSD%, n = 6)	Mean Recovery(%) (n = 6)	
			Intracellular	Extracellular
ZEN	1.29	0.96	102.13	91.41
Z14G	1.35	0.55	100.81	100.73
α-ZEL	0.86	1.11	88.03	92.59
β-ZEL	0.91	0.98	109.83	85.58

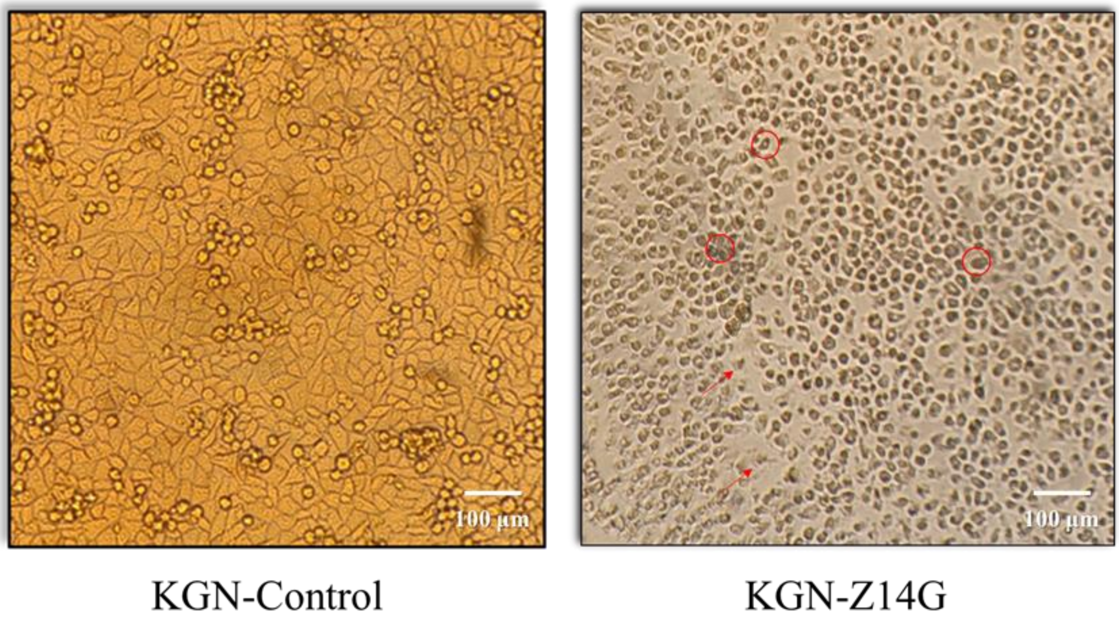

Figure S1. Cell Morphology of KGN after Administration of Z14G (420 µM, 24 h) (100×), red circles represent cell shrinkage and red arrows represent cell fragmentation.
